# PB.11. Modern management of acute breast abscesses: radiological interventions replacing surgical incisions?

**DOI:** 10.1186/bcr3731

**Published:** 2014-11-03

**Authors:** N Healy, M Stenson, S Harte

**Affiliations:** 1 St James Hospital, Dublin, Ireland

## Introduction

Traditionally acute breast abscesses were managed with a combination of antibiotics and surgical intervention. Despite advances in imaging techniques and minimally invasive interventions, acute abscesses remain poorly managed with significant treatment delays. The aim of this study was to review management of acute breast abscesses in a symptomatic breast service over 4 years and to develop an updated algorithm for effective and minimally invasive abscess management.

## Methods

From January 2010 to June 2014 all acute abscesses referred to the radiology service were retrospectively reviewed with attention to patient demographics, US findings, aetiology, radiological or surgical intervention, treatment duration and outcome. Total number of ultrasounds (USs), aspirations and US-guided catheter placements were recorded.

## Results

A total of 203 acute abscesses attended with 160 US-guided aspirations and 43 US-guided catheter placements over 4 years. Patients required on average 2.3 US and 1.4 aspirations during each acute episode. Puerperal abscesses accounted for 38 (23%) of the aspiration cases and for 29 (67%) of the catheter cases. The mean abscess size managed with drainage catheters and aspiration was 4.4 cm and 2.7 cm respectively. Four (2.2%) patients ultimately required surgical intervention.

## Conclusions

Radiological assessment and minimally invasive intervention is an accessible and effective strategy in the management of acute breast abscesses. This approach limits more aggressive surgical interventions with improved patient acceptability. An updated treatment algorithm should be adopted in all symptomatic breast clinics to ensure timely treatment and optimise outcome.

